# Development of the binaural intelligibility level difference for speech-in-speech recognition

**DOI:** 10.1121/10.0039675

**Published:** 2025-10-21

**Authors:** Lori J. Leibold, Jenna Felder, Elizabeth Benson, Emily Buss

**Affiliations:** 1 Center for Hearing Research, Boys Town National Research Hospital, Omaha, Nebraska 68131, USA; 2 MED-EL US, Durham, North Carolina 27713, USA; 3 Baker Audiology and Hearing Aids, Sioux Falls, South Dakota 57108, USA; 4 Department of Otolaryngology/Head and Neck Surgery, University of North Carolina at Chapel Hill, Chapel Hill, North Carolina 27599, USA lori.leibold@boystown.org, Jenna.Felder@medel.com, libby@siouxfallshearing.com, ebuss@med.unc.edu

## Abstract

This study aimed to characterize maturation of the binaural intelligibility level difference (BILD) for school-age children in the context of speech-in-speech recognition. Children (5–17 years) and adults completed an adaptive, open-set sentence recognition task in a two-talker masker in two binaural conditions: (1) target and masker speech in-phase across the two ears, and (2) target speech presented 180° out-of-phase across the two ears and masker speech presented in-phase. Estimates of the BILD, computed as the difference score between the two binaural conditions, were mature by ten years of age, consistent with previous data on the BILD for speech-in-noise recognition.

## 1. Introduction

Masked speech recognition thresholds (SRTs) in a diotic noise masker are lower when target speech is presented out-of-phase across the two ears (i.e., M_0_T_π_) relative to when target speech is presented in-phase across the two ears (i.e., M_0_T_0_). This effect is often referred to as the binaural intelligibility level difference (BILD; Licklider, 1948), reflecting a benefit of binaural difference cues for speech recognition. The average magnitude of the BILD for adults with normal hearing ranges from 4–8 dB across studies for recognition of sentences (e.g., Goverts and Houtgast, 2010), digits (e.g., De Sousa *et al.*, 2020), and words (e.g., Merchant *et al.*, 2021; Wilson *et al.*, 1982) presented in speech-shaped noise.

School-age children take advantage of binaural phase differences in the context of speech-in-noise recognition, although findings from several studies suggest the magnitude of the BILD is reduced in children younger than nine to ten years of age (Koopmans *et al.*, 2018; Wolmarans *et al.*, 2021; Neher *et al.*, 2022; Dunlap *et al.*, 2024; but see Merchant *et al.*, 2021). For example, Koopmans *et al.* (2018) reported estimates of the BILD for four- to ten-year-old children and 18- to 30-year-old adults with normal hearing. Participants completed testing in M_0_T_0_ and M_0_T_π_ conditions using the digits-in-noise (DIN) test (Smits *et al.*, 2013), which relies on an adaptive procedure to estimate the SRT corresponding to 50% correct recognition of digit triplets presented in speech-shaped noise. SRTs improved with increasing child age in both conditions, with adult-like performance not observed until ten years of age. SRTs improved more rapidly with increasing child age in the M_0_T_π_ compared with the M_0_T_0_ condition, resulting in average BILDs of 3 dB for four- to six-year-olds, 4 dB for seven- to nine-year-olds, and 5 dB for ten- to 12-year-olds and adults.

In natural listening environments such as classrooms, children are often tasked with hearing and understanding speech produced by one talker (e.g., the teacher) when multiple people are talking at the same time (e.g., classmates). Thus, assessing the benefits of binaural hearing for speech-in-speech recognition could shed light on children's real-world speech perception. However, only a handful of studies have reported the BILD for speech-in-speech recognition for children or for adults. The available data collected on adults suggest that the magnitude of the BILD is either similar (Phatak *et al.*, 2018; Calandruccio *et al.*, 2020) or smaller (Carhart *et al.*, 1969; Goverts *et al.*, 2007) for speech-in-speech relative to speech-in-noise recognition. This is perhaps surprising given reports that the binaural difference cue facilitates segregation of target from masker speech (e.g., Lavandier and Culling, 2010). One consideration is that the specific combinations of target and masker speech used in previous studies investigating the BILD for speech-in-speech recognition may have produced relatively small amounts of informational masking. For example, SRTs in M_0_T_0_ conditions for adults tested by Goverts *et al.* (2007) were substantially lower (better performance) when the masker was a single stream of speech relative to stationary noise. Higher levels of informational masking tend to be observed with two competing talkers relative to a single talker (e.g., Freyman *et al.*, 2004), which in turn may increase the magnitude of the BILD.

Previous investigations reporting estimates of the BILD for children in the context of speech-in-speech recognition are based on relatively small samples of children falling within a narrow age range (Bornstein and Musiek, 1992; Calandruccio *et al.*, 2020). Calandruccio *et al.* (2020) investigated the extent to which four- to seven-year-old children and young adults benefit from clear speech modifications when listening to sentences in a two-talker masker. Participants in Experiment 2 of their study completed testing in M_0_T_π_ and M_0_T_0_ conditions. The average BILD for target speech produced using a conversational speaking style was smaller for children (4.4 dB) than for adults (6.8 dB), with a trend for increasing binaural benefit with increasing age in the child sample.

The goal of this study was to characterize age-related changes in the BILD across the school-age years in the presence of two streams of competing speech produced by talkers matched in sex to the target talker. SRTs for masked sentence recognition were estimated adaptively for each participant in M_0_T_0_ and M_0_T_π_ conditions. Given prior research investigating age-related changes in masked speech recognition (e.g., Hall *et al.*, 2002; Corbin *et al.*, 2016), it was predicted that SRTs would improve with increasing child age until adolescence for both M_0_T_0_ and M_0_T_π_ conditions. If the BILD in young children is limited solely by binaural processing, then we would expect to see a similar result for speech recognition in competing speech as observed in noise, namely, mature BILD estimates around ten years of age (e.g., Koopmans *et al.*, 2018; Wolmarans *et al.*, 2021; Neher *et al.*, 2022). Alternatively, informational masking associated with the use of a two-talker speech masker could tax young listeners' abilities to segregate and/or selectively attend to the target, resulting in a more prolonged time-course of maturation for the BILD than observed previously for speech-in-noise recognition.

## 2. Methods

### 2.1 Participants

A total of 30 adults (19.0 to 37.8 years; mean age = 26.6 years) and 77 children (5.3 to 17.6 years of age; mean age = 10.1 years) participated in this study. Inclusion criteria were (1) native speaker of American English; (2) no recent ear infections or history of chronic middle ear disease, by self-or parent report; and (3) passed a hearing screening prior to testing (i.e., thresholds less than or equal to 20 dB hearing level (HL) for octave frequencies between 250 and 8000 Hz; ANSI, 2018).

### 2.2 Stimuli and conditions

Target stimuli were revised Bamford-Kowal-Bench (BKB) sentences (see Bench *et al.*, 1979). The revised BKB corpus contains 21 lists of 16 sentences. This corpus uses simple vocabulary and syntax, and normative data for children ≥5 years of age have been established (e.g., Holder *et al.*, 2016). Each sentence contains three or four key words, resulting in a total of 50 keywords per list. The sentences were spoken by an adult female talker who is a native speaker of American English. Sentence recognition was assessed in a two-female-talker masker, described in detail by Calandruccio *et al.* (2014). The masker sample was repeated without discontinuity during testing. A sampling rate of 44 100 Hz was used for all speech recordings. This rate was reduced to 24 414 Hz for the present experiment.

Participants were tested in two binaural conditions: (1) M_0_T_0_ in which both the target and masker speech were presented in-phase across ears, and (2) M_0_T_π_ in which target speech was presented 180° out-of-phase across the two ears and the masker speech was presented in-phase. A custom matlab script was used to select the test conditions and present the stimuli. Target and masker stimuli were processed using a real-time processor (RZ6, Tucker Davis Technologies, Alachua, FL) and presented via headphones (HD 25-1 II, Sennheiser, Wedemark, Germany).

### 2.3 Procedure

SRTs were measured for each condition using an open-set sentence recognition procedure. Participants wore headphones and sat inside a single-walled sound-treated booth facing a window. A microphone (GSI-61 talkback microphone, Grason-Stradler, Eden Praire, MN) mounted above the booth window routed the participant's verbal responses to an audiometer (Grason-Stradler, GSI-61). An examiner sitting outside of the booth wore a headset (Grason-Stradler, GSI-61 operator's headset) and scored the participant's verbal responses in real time. The talkback volume on the audiometer was adjusted as needed to give the examiner a clear, audible signal. The examiner was able to view the participant's face through the window.

Participants were instructed to ignore the competing speech background and to verbally repeat the sentences that they heard. Participants were informed that they were scored on a word-by-word basis and should repeat as many words as they heard. They were encouraged to guess if they were unsure. Keywords were scored correct if the entire word was correctly repeated. If the participant did not respond, the examiner waited approximately 5 s before marking all keywords as incorrect. The participant was not provided with feedback.

An adaptive tracking procedure was used to characterize individual psychometric functions. The level of the masker was fixed at 60 dB sound pressure level (SPL) (57 dB SPL for each of two speech streams). The level of target sentences was adaptively varied using a pair of interleaved tracks, following a 1-down, 1-up stepping rule. One track used a strict criterion for scoring the sentence correct (one or fewer keywords wrong), and the other used a lax criterion (one or more keywords right). For each track, an initial step size of 8 dB was used, which reduced to 4 dB after the first reversal and then to 2 dB after the second reversal. Each run stopped after 64 sentences were presented (four lists, each with 16 sentences). Two-parameter logit functions were fitted to the word-level data obtained for each participant in each condition, and the target-to-masker ratio (TMR) corresponding to the 50% correct point was estimated based on these fits.

Sentence lists were randomized across participants and conditions, and no sentences were repeated for a given participant. Sentence recognition testing took approximately 30 min to complete, including breaks.

### 2.2 Statistical analyses

Statistical analyses were conducted in SPSS (version 29.0.0). Child age was represented on a log_10_ scale prior to data analysis, based on observations that the rate of development for masked speech recognition is greater for younger than for older school-age children (e.g., Buss *et al.*, 2017).

## 3. Results

**Fig. 1.**
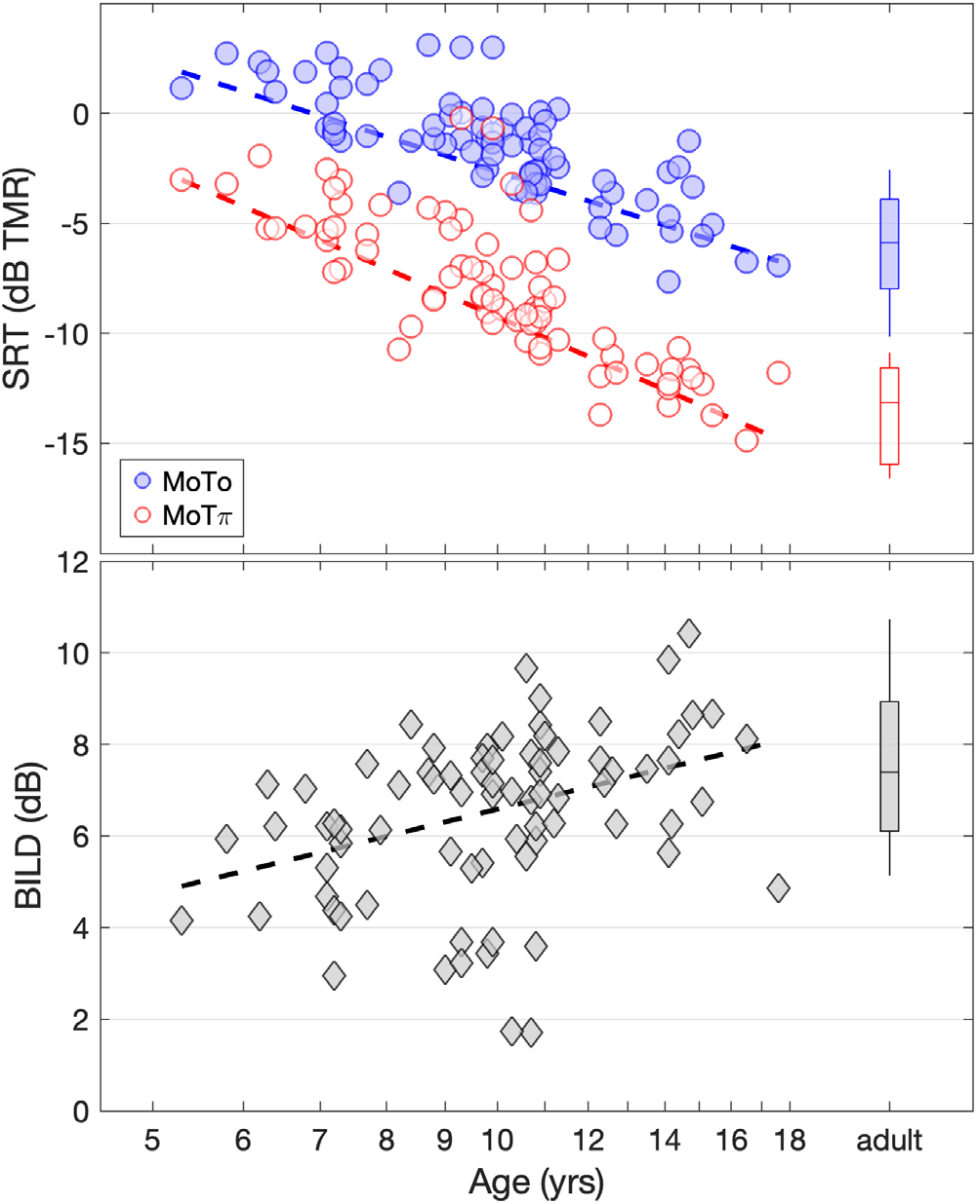
The top panel shows speech recognition thresholds (SRTs) for individual children as a function of age for M_0_T_0_ (filled circles) and M_0_T_π_ (open circles) conditions. Boxplots at the right of the panel show the distribution of SRTs for adults for M_0_T_0_ (filled box) and M_0_T_π_ (open box) conditions. Regression lines fitted to the child data are shown for both conditions. The bottom panel shows estimates of the BILD (M_0_T_0_–M_0_T_π_) for individual child participants as a function of child age. Positive values indicate better performance when target sentences were presented out-of-phase across ears (M_0_T_π_) compared to when the target sentences were presented in-phase across ears (M_0_T_0_). The distribution of BILD estimates for adults is shown on the right of the panel. A regression line is fitted to the child data.

Psychometric functions fitted to the data of individual participants accounted for more than 50% of the variance in all cases, with a median of 90% of variance accounted for across participants (range = 54% to 100%). The SRTs for individual children are shown in Fig. 1 (top), plotted as a function of child age on a log scale. Filled and open circles indicate SRTs in the M_0_T_0_ and M_0_T_π_ conditions, respectively. The distribution of SRTs for adults in each condition is shown on the right.

There was a trend for better speech-in-speech recognition performance with increasing age during childhood for both binaural conditions. Results of a linear regression model conducted on the child data confirmed this observation. The dependent variable was SRT, and the independent variables were log-transformed age, binaural condition, and the interaction between age and condition. The M_0_T_π_ condition served as the reference, and age was mean centered prior to running the model. There were no indications of violations of normality assumptions for the model based on residual assessment. This model provided a statistically significant fit to the data (*R*^2^ = 0.83), indicating significant effects of age (*β* = −16.72, *t* = −8.98, *p* < 0.001), binaural condition (*β* = −6.41, *t* = −21.91, *p* < 0.001), and a significant interaction between age and binaural condition (*β* = 6.12, *t* = 2.33, *p* = 0.021).

Across the age range of children tested (from 5.3 to 17.6 years), model predictions indicated an improvement in SRT of 8.7 and 11.9 dB for M_0_T_0_ and M_0_T_π_ conditions, respectively. Estimates of mature performance were based on the age at which the mean child SRT falls within the 95% confidence interval of the adult data. This approach predicts mature performance by 15.6 years for the M_0_T_0_ condition and 14.9 years for the M_0_T_π_ condition.

Estimates of the BILD, computed for each participant as the difference in SRTs between the two conditions (M_0_T_0_*–*M_0_T_π_), are also shown as a function of age for individual child listeners in Fig. 1 (bottom). The distribution of adult BILD estimates is shown on the right. Positive estimates indicate better performance in the M_0_T_π_ condition relative to the M_0_T_0_ condition. All child participants and all but one adult participant^1^ benefited from the interaural difference in the M_0_T_π_ condition, as demonstrated by a positive BILD. Across the age range of children tested (from 5.3 to 17.6 years), model predictions indicated an increase in the BILD of 3.2 dB. The magnitude of the BILD was ≥3 dB for 75 (out of 77) children and 29 (out of 30) adults who participated in this experiment. Considerable overlap between BILD estimates for children and adults was observed. The mean BILD for adults was 7.3 dB [standard deviation (SD) = 2.66]. More than half (44/77) of the BILD estimates for children fell within the 95% confidence interval (CI) around the mean for adults. A line fit to child data predicts the mean BILD within the 95% confidence interval of adult data by 9.5 years of age.

Although the maturation of SRTs is often fitted with a line, there are reasons to suspect nonlinearity, particularly when testing children over a wide age range. For example, SRTs for speech-in-speech recognition are sometimes observed to hover around zero dB TMR at the low end of the age range (Corbin *et al.*, 2016), which could reflect failure to segregate the target and masker in the youngest listeners, and they may reach a lower asymptote at the upper end of the child age range, representing mature performance. Possible effects of an upper and/or lower asymptote can be characterized by fitting a logit function that is scaled and shifted to capture the range of SRTs observed (four parameters total). Fitting a logit to SRTs measured from children in the present study results in very small improvements compared to line fits, increasing the percentage of variance accounted for by ≤ 1.7% points (refer to supplementary material Fig. 1). This result lends support to the use of linear models to characterize maturation of SRTs in the present dataset.

## 4. Discussion

In this study, school-age children and adults completed a sentence recognition task in the presence of two competing streams of speech which were expected to produce substantial informational masking. SRTs for each participant were estimated in M_0_T_π_ and M_0_T_0_ conditions. Results in both conditions reinforce prior observations that the ability to recognize speech in a two-talker masker follows a prolonged time course of development that extends into adolescence (e.g., Corbin *et al.*, 2016; Leibold *et al.*, 2024), with adult-like performance by 14.9 (M_0_T_π_) and 15.6 (M_0_T_0_) years of age. The maturation of speech-in-speech recognition using stimuli like those employed in the present study is thought to involve central auditory and cognitive processes needed to separate and selectively attend to target speech in a speech masker, as well as the development of language and vocabulary (e.g., Corbin *et al.*, 2016; Sanchez *et al.*, 2022; Lalonde *et al.*, 2024).

Adult-like BILD estimates were observed by 9.5 years of age for sentence recognition in a two-talker speech masker. This pattern of development aligns closely with previous studies of school-age children investigating the BILD for speech-in-noise recognition (e.g., Koopmans *et al.*, 2018; Wolmarans *et al.*, 2021; Neher *et al.*, 2022). For example, Wolmarans *et al.* (2021) reported an increase in the magnitude of the BILD until 9.9 years of age based on M_0_T_π_ and M_0_T_0_ conditions of the DIN test. The consistency of age effects in the BILD across studies despite differences in stimuli and task demands provides converging evidence that the ability to capitalize on interaural phase differences to recognize masked speech is mature by about ten years of age.

Koopmans *et al.* (2018) posited that the age-related improvements in the BILD observed in the context of the DIN test reflect maturation of binaural processing skills. The DIN test was designed to reduce the impact of non-auditory factors on performance (Smits *et al.*, 2013), and subsequent studies involving adults provide evidence that individual differences in language and cognitive skills are not associated with performance on the DIN test (Kaandorp *et al.*, 2016; Heinrich *et al.*, 2015). In contrast, the target sentences, two-talker masker, and open-set recognition procedure utilized in the present study are likely to increase the linguistic and cognitive demands placed upon the listener (e.g., McCreery *et al.*, 2020). Finding a similar developmental trajectory for DIN and sentences in two-talker speech lends further support to the idea that maturation of the BILD reflects development of binaural processing as opposed to more cognitive or linguistic abilities.

The broader literature on binaural hearing evaluated with other paradigms is only partly consistent with this result, however, in that some data indicate mature or nearly mature performance in infancy or early childhood (e.g., Lovett *et al.*, 2012), and others indicate continued maturation into adolescence (e.g., Kuhnle *et al.*, 2012). For example, some data show that sensitivity to interaural time differences (ITDs) is mature or nearly mature by ten years of age (Ehlers *et al.*, 2016; Flanagan *et al.*, 2021). However, other data suggest that this might not be the case (van Deun *et al.*, 2009; Syeda *et al.*, 2023), with maturation of both auditory working memory and listening strategy implicated in ITD processing (Peng *et al.*, 2020; Syeda *et al.*, 2023). Because the BILD is a difference score, it may be less susceptible to domain general immaturity (e.g., increased lapses of attention) than measures based on a single score. However, spatial release from masking (SRM) is also based on a difference score**—**performance for collocated vs spatially separated talkers**—**and there is variability in the age of maturity for this paradigm too; some studies report mature performance in late childhood or early adolescence (e.g., Cameron *et al.*, 2011; Yuen and Yuan, 2014; Corbin *et al.*, 2017), and others report adult-like performance in early childhood (Litovsky, 2005).

In the present dataset, estimates of the BILD were ≥3 dB for 77/79 children and 29/30 adults, suggesting that children as young as five years of age with normal hearing take advantage of binaural cues for recognizing speech in a speech masker. These findings support the potential for using the BILD paradigm for evaluating binaural hearing in children when it is not feasible to use physically separated sound sources,^2^ with the caveat that estimates of the BILD and SRM may not reflect exactly the same binaural abilities (Neher *et al.*, 2022). Potential applications include monitoring the binaural hearing abilities of children with chronic otitis media (Merchant *et al.*, 2021), differentiating types of hearing loss (e.g., De Sousa *et al.*, 2022), and confirming that participants wore headphones as instructed during remote online testing (Woods *et al.*, 2017).

## 5. Conclusion

In conclusion, these results indicate that the BILD for sentence recognition in a two-talker masker increases with age during childhood. Whereas SRTs for sentences in two-talker speech do not reach adult levels until around 15 years of age, mature estimates of BILD were observed around 9.5 years of age, consistent with prior data on the BILD for speech in noise. Nearly all children and adults in the study had a BILD of 3 dB or greater, suggesting that children as young as five years of age with normal hearing can take advantage of binaural cues for recognizing speech in a speech masker.

## Supplementary material

See the supplementary material for data fitted using a 4-parameter logit function. SRTs for individual children are plotted as a function of age for M_0_T_0_ (filled circles) and M_0_T_π_ (open circles) conditions.

## Data Availability

The data that support the findings of this study are available from the corresponding author upon reasonable request.

## Footnotes

^1^
The BILD estimate for this participant was −0.6 dB TMR.

^2^
There are other alternatives to physically separated sound sources, such as simulating virtual sound sources under headphones with head-related transfer functions (e.g., the LiSN test; Cameron and Dillon, 2007). One possible advantage of the BILD is that it is simple and inexpensive to implement.
